# BAP31 regulates IRAK1-dependent neuroinflammation in microglia

**DOI:** 10.1186/s12974-019-1661-7

**Published:** 2019-12-28

**Authors:** Xia Liu, Kun Jiao, Cong-cong Jia, Guo-xun Li, Qing Yuan, Ji-kai Xu, Yue Hou, Bing Wang

**Affiliations:** College of Life and Health Science, Northeastern University, 195 Chuangxin Road, Hunnan District, Shenyang, Liaoning 110819 People’s Republic of China

**Keywords:** Microglia, BAP31, Neuroinflammation, IRAK1, Memory deficiency

## Abstract

**Background:**

Microglia, the mononuclear immune cells of the central nervous system (CNS), are essential for the maintenance of CNS homeostasis. BAP31, a resident and ubiquitously expressed protein of the endoplasmic reticulum, serves as a sorting factor for its client proteins, mediating the subsequent export, retention, and degradation or survival. Recently, BAP31 has been defined as a regulatory molecule in the CNS, but the function of BAP31 in microglia has yet to be determined. In the present study, we investigated whether BAP31 is involved in the inflammatory response of microglia.

**Methods:**

This study used the BV2 cell line and BAP31 conditional knockdown mice generated via the Cre/LoxP system. A BAP31 knockdown experiment was performed to elucidate the role of BAP31 in the endogenous inflammatory cytokine production by microglial BV2 cells. A mouse model of lipopolysaccharide (LPS)-induced cognitive impairment was established to evaluate the neuroprotective effect of BAP31 against neuroinflammation-induced memory deficits. Behavioral alterations were assessed with the open field test (OFT), Y maze, and Morris water maze. The activation of microglia in the hippocampus of mice was observed by immunohistochemistry. Western blot, enzyme-linked immunosorbent assay (ELISA), immunofluorescence staining, and reverse transcription quantitative real-time polymerase chain reaction (RT-PCR) were used to clarify the mechanisms.

**Results:**

BAP31 deficiency upregulates LPS-induced proinflammatory cytokines in BV2 cells and mice by upregulating the protein level of IRAK1, which in turn increases the translocation and transcriptional activity of NF-κB p65 and c-Jun, and moreover, knockdown of IRAK1 or use of an IRAK1 inhibitor reverses these functions. In the cognitive impairment animal model, the BAP31 knockdown mice displayed increased severity in memory deficiency accompanied by an increased expression of proinflammatory factors in the hippocampus.

**Conclusions:**

These findings indicate that BAP31 may modulate inflammatory cytokines and cognitive impairment induced by neuroinflammation through IRAK1, which demonstrates that BAP31 plays an essential role in microglial inflammation and prevention of memory deficits caused by neuroinflammation.

## Background

Neuroinflammation has been implicated in the etiology of most neurodegenerative diseases, including Alzheimer’s disease [1], Parkinson’s disease, and schizophrenia [2]. Preclinical and clinical studies have established that neuroinflammation is not merely a response to pathophysiological events, but also contributes to and drives pathogenesis [3]. Microglia, resident inflammatory cells, play a decisive role in neuroinflammation, which generally involves ramified processes for communication and surveillance the environment. When activated, microglia can perform many diverse functions that may be either beneficial or detrimental depending on the nature of the initial stimulus.

Lipopolysaccharide (LPS), a major bacterial Toll-like receptor 4 (TLR4) ligand, can trigger an innate immune response, induce neuroinflammation, and influence the function of neuronal cells, thus leading to cognitive impairment. Intracerebroventricular administration of LPS is a well-established model of cognitive and behavioral impairment. Neuroinflammation gives rise to memory impairment [4]. The level of amyloid-β (Aβ), and the activities of β- or γ-secretases are increased in the hippocampus upon LPS administration [5]. Acute neuroinflammation impairs context discrimination memory and disrupts pattern separation processes in the hippocampus [6].

Pathogen-associated molecular patterns (PAMPs) are recognized by pattern recognition receptors (PRRs) and result in the production of proinflammatory cytokines. Toll-like receptors are vital members of PRRs, and when ligands such as LPS are engaged, there is an interaction with the myeloid differentiation factor 88 (MyD88) TIR domain, then MyD88 recruits IL-1R-associated kinase 4 (IRAK4) and promotes the phosphorylation of IL-1 receptor-associated kinase (IRAK1). IRAK1 subsequently interacts with tumor necrosis factor receptor-associated factor 6 (TRAF6) and TAK1 kinase, which activate the IκB kinase complex to phosphorylate and promote proteasomal degradation of IκB protein, an inhibitor of the transcription factor nuclear factor kappa B (NF-κB). Moreover, TAK1 can activate JNK and p38 mitogen-activated protein kinase (MAPK) family members, which trigger AP-1 activation [7].

B cell receptor-associated protein 31 (BAP31), encoded by BCAP31, is an integral polytopic endoplasmic reticulum (ER) membrane protein with three transmembrane domains [8]. Previous studies have shown that BAP31 is mainly involved in the activation of B cells [9], is a carrier protein that transports membrane proteins [10, 11] from the endoplasmic reticulum, and is associated with apoptosis [12, 13] and tumors [14]. Recently, BAP31 was also reported to function as a regulatory molecule for immunity in the central nervous system (CNS); mutation of BAP31 causes X-linked syndrome, including motor and intellectual disabilities, congenital microcephaly, dystonia, sensorineural deafness, and white matter changes [15].

Our previous research found that BAP31 was involved in T cell activation through the T cell antigen receptor (TCR) signaling pathway [16]. BAP31deficiency contributes to the formation of amyloid-β plaques [17]. Considering that BAP31 is widely expressed in glial cells, however, the effect of BAP31 in the central nervous system is not well understood. Therefore, in this study, we aimed to investigate the role of BAP31 in LPS-mediated signaling and inflammation mediator expression by knocking down BAP31 in BV2 microglial cells. BAP31 knockdown resulted in increased IRAK1 protein expression. Consequently, BAP31 deficiency increased the transcriptional activity of NF-κB p65 and c-Jun and augmented their translocation from the cytoplasm to the nucleus, resulting in increased inflammatory cytokines.

We found for the first time that BAP31 deficiency exacerbates the activation of microglia and the death of neurons induced by LPS. This issue sheds light on how BAP31 influences the activation of microglia, and suggests that BAP31 may be therapeutic by stabilizing the activity of the IRAK1 and LPS-derived cytokines, which might effectively interfere with the progression of neuroinflammation.

## Materials and methods

### Animals

Details on the targeting construct and targeting procedure were reported in our previous study [18–20]. BAP31^fl/fl^ (with the BAP31 allele floxed at exon 3) C57BL/6 mice were mated with the transgenic C57BL/6 mice carrying a Cre recombinase with LysM promoter. Mice with a BAP31 deletion in microglia were obtained by crossing Cre ^LysM-/-^ BAP31^fl/fl^ mice strain and Cre ^LysM+/-^ BAP31^fl/fl^ mice strain. The genotype of the mice was analyzed by PCR of tail DNA using primers: BAP31^fl/fl^ primers: sense 5′-GCCTCTACAGGATGCTCATTT-3′; antisense 5′-GGACAGTCATGCTAG TCTG AGG-3′; Cre primers: sense 5′-GAGACTCTGGCTACTCATCC-3′; and antisense 5′-CCTTCAGCAAGAGCTGGGGAC-3′. All experiments were performed with mice aged 8–10 weeks. Mice were group-housed in cages of three to five in a 12 h light/dark cycle with food and water provided ad libitum. All experimental procedures were critiqued and approved by the Committee of Experimental Animal Adiministration of Northeastern University in accordance with the National Institutes of Health’ Guidelines for the Care and Use of Laboratory Animals.

### Antibodies and chemical reagents

Anti-p-TAK1 (9339), anti-TAK1 (5206), anti-p-P38 (9211), anti-P38 (9212), anti-p-JNK (9251), anti-JNK (9252), anti-p65 (8242) anti-p-p65 (3033), anti-IκBα, anti-p-IKKα/β (14938), anti-IRAK1 (4504), and anti-MyD88 (4283) were from Cell Signaling Technology (Danvers, MA, USA); Dulbecco’s modified Eagle medium (DMEM), fetal bovine serum (FBS), and 0.25% trypsin were purchased from Gibco BRL (Grand Island, NY, USA). 3-[4,5-Dimethyl thiazol-2-yl]-2,5-diphenyltetrazolium bromide (MTT), lipopolysaccharide (LPS), tumor necrosis factor α (TNFα), and IRAK1/4 inhibitor (C20H21N5O4) were purchased from Sigma Chemical Co. (St. Louis, MO, USA); IL-1β and TNFα enzyme-linked immune sorbent assay (ELISA) kit were from R&D systems (Minneapolis, MN, USA).

### MTT assay

Scramble BV2 microglial cells and shBAP31 BV2 microglial cells were plated at 1 × 10^4^ cells/well in 96-well plates. After incubation overnight, the cells were treated with or without LPS (100 ng/ml) for 24 h, 20 μl MTT (5 mg/ml in PBS) solution was added to each well and incubated for 4 h, the supernatant was removed, and 150 μl dimethyl sulfoxide (DMSO) was added to solubilize the formazan crystals. The absorbance was measured at 490 nm using a multimode microplate reader (Bio-Tek, USA).

### Nitrite assay

Accumulation of nitrite (NO_2_^−^) in culture supernatant fluids was measured by the Griess assay. Microglial cells (5 × 10^4^ cells/well) were plated into 96-well plates, then treated with LPS (100 ng/ml) for 24 h. Then, 50 μl culture supernatant fluids were mixed with 50 μl Griess reagent at 37 °C. Fifteen minutes later, the absorbance was determined at 540 nm.

### siRNA and cell transfection

IRAK1 siRNA and control siRNA were purchased from Gene Pharma (Shanghai, China). The following sequences were used, IRAK1-mus-916: sense 5′-UAGAG UGGACUAUGGUGAATT-3′, antisense 5′-UUCACCAUAGUCCACUCUATT-3′; IRAK1-mus-1583: sense 5′-CUGCCCAGAUCUAUAAGAATT-3′, antisense 5′-UUCUUAUAGAUCUGGGCAGTT-3′; IRAK1-mus-858: sense 5′-CGAGCAGUCA UGAGAAAUATT-3′, antisense 5′-UAUUUCUCAUGACUGCUCGTT-3′; and control siRNA: sense 5′-UUCUCCGAACGUGUCACGUTT-3′, antisense 5′-ACGU GACACGUUCGGAGAATT-3′. Cells were seeded at 2 × 10^4^ cells/well in 6-well plates. After incubation overnight, the cells were transfected with 50 nmol/l IRAK1 siRNA using Lipofectamine 3000 reagent for 60 h according to the manufacturer’s protocol. Then, the cells were exposed to LPS for 30 min, and the nuclei were separated from the cytoplasm.

### BAP31 shRNA and transfection

The pL/shRNA/Green fluorescent protein (GFP)-mouse-BAP31 (shBAP31) lentiviral construct and control construct pL/shRNA/GFP (Scramble) were purchased from Novobio Technology (Shanghai, China). The following sequences were used sense 5′-CACCG*CCATGGCTTATAGATCATTATCGAAATAATGATCTATAAG CCATGG-3′ and antisense 5′-AAAACCATGGCTTATAGATCATTATTTCGATA AGATCTATAAGCCATGGC*-3′. BV2 cells were grown for 24 h, infected with shBAP31 and scramble lentiviral constructs (multiplicity of infection = 250) at 37 °C for 72 h, then selected using 0.4 μg/ml blasticidin to screen single cell clones for 2 weeks and expanded in culture for 4 weeks. The knockdown efficiency was measured using Western bloting and reverse transcription quantitative real-time polymerase chain reaction (RT-PCR).

### Enzyme-Linked Immunosorbent assay

The levels of interleukin-1β (IL-1β) and TNFα in the conditioned medium were measured by ELISA kits according to the manufacturer’s instructions (R&D Systems, Minneapolis, MN).

### Primary microglial cell culture

Primary microglial cells isolated from postnatal mice born within 24 h. Briefly, brains were isolated in DMEM/F12 medium supplemented with 10% FBS and 1% penicillin/streptomycin and stripped of olfactory bulbs, cerebellum, and midbrain, and meninges were removed. Brain tissue was digested using trypsin and resuspended in DMEM-F12 medium. Cell suspensions were incubated in 25 cm^2^ flasks pretreated with poly-l-lysine. After 4–7 days, astrocytes recovered and microglia are generated by addition of DMEM medium containing 25% of L929 conditioned medium. Three to 4 days later, microglia were isolated from mixed glial cell cultures by shaking at 100 rpm for an hour. Microglia were resuspended in RPMI containing 25% L929 conditioned medium.

### Brain histology and immunofluorescence

The mice were perfused with saline and 4% paraformaldehyde under deep anesthesia, the brains were fixed in 4% paraformaldehyde for 24 h at 4 °C, fully dehydrated in 30% sucrose solution, and then embedded (*n* = 12 per group for each experiment). The frozen brains were cut into 10-μm-thick slices using a microtome blade (Leica, Wetzlar, HE, Germany). Endogenous peroxidase activity was blocked with 0.3% H_2_O_2_ for 10 min, and washed in PBS, blocked for 1 h in 5% BSA, then species were incubated with the primary antibody overnight at 4 °C. After 3 × 5 min washes with PBS solution, specimens were incubated with secondary antibody (Alexa Fluor 488 or 568; Invitrogen Carlsbad, CA) for 2 h at room temperature. Sections were then washed in PBS, and used to visualize immunoreactivity. Sections were viewed and processed in a Leica (Wetzlar, HE, Germany) scanning confocal microscope. Ionized calcium binding adapter molecule 1 (Iba1)/NeuN-positive cells were counted using ImageJ software (NIH) with a DAPI counterstain. The average number of cells/field of view was used for statistical analysis.

### Immunoblotting

Cells were harvested and lysed in RIPA lysis buffer (1mol/L Tris-HCl, pH 7.4; 1% Triton-X-100; 1% sodium deoxycholate; 150 mM NaCl 0.1% SDS) with protease and phosphatase inhibitor cocktails and 1 mM phenylmethanesulfonyl fluoride (PMSF). The samples were centrifuged at 12,000×*g* for 15 min at 4 °C. Protein content was measured by the micro-BCA protein assay kit (Thermo Fisher Scientific, Wal- tham, MA, USA). Equal amounts of total proteins lysates were then separated by 12% SDS-PAGE and transferred to Immobilon polyvinylidene difluoride (PVDF) membranes (Millipore). The membranes were subsequently blocked with 5% nonfat milk in TBST (Tris-buffered saline: 20 mM Tris-HCl; 137 mM NaCl; 0.1% Tween-20; pH 7.6) and probed with primary antibodies, followed by treatment with HRP-linked secondary antibodies and ECL Western blotting detection reagents. The intensity of immune-reactive bands were quantified using Image Lab software.

### RNA extraction, reverse transcription, and quantitative real-time PCR

Total RNA was isolated from cells using TRIzol reagent (Carlsbad, CA, USA), and reverse transcription was performed using GoScript^TM^ Reverse Transcription System (Promega, Madison, USA). Quantitative real-time PCR (qRT-PCR) was performed using Go Taq® qPCR Master (Promega, Madison, USA). The relative fold change in the expression of each messenger RNA (mRNA) was calculated using the ΔΔCt method relative to the expression of GAPDH. PCR primers for IL-1β, TNFα, COX2, MyD88, IRAK1, TLR4, and GAPDH were designed as follows: IL-1β primers sense 5′-TGACGGACCCCAAAAGATGA-3′; antisense 5′-TCTCCACAGCCACA ATGAGT-3′; TNFα primers sense 5′-CCCTCACACTCAGATCATCTT CT-3′; antisense 5′-GCTACGACGTGGGCTACAG-3′; COX2 primers sense 5′-TGCATTC TTTGCCCAGCACT-3′; antisense 5′-AAAGGCGCAGTTTACGCTGT-3′; MyD88 primers sense 5′-CCGGAACTTTTCGATGCCTT-3′; antisense 5′-AGAAACAACCA CCACCATGGC-3′; IRAK1 primers sense 5′-TTCCACTCCCTGTTTCCCTC-3′; antisense 5′-AACCACCCTCTCCAATCCTG-3′; TLR4 primers sense 5′-TCTGGG GAGGCACATCTTCT-3′; antisense 5′-AGGTCCAAGTTGCCGTTTCT-3′; GAPDH forward sense 5′-AGGTCGGTGTGAACGGATTTG-3′; antisense 5′-TGTAGACCATGTAGTTGAGGTCA-3′.

### Luciferase reporter assay

Luciferase reporter assay was performed as previously described. Raw264.7 and HEK293T scramble and shBAP31 cells were cotransfected with NF-κB luciferase reporter plasmid or AP-1 luciferase reporter plasmid and Renilla luciferase plasmid (pRL-SV40-C) using Lipofectamine 3000 reagent (Invitrogen) for 48 h according to the manufacturer’s instructions. Then, Raw264.7 cells were stimulated with LPS (1 μg/ml) for 24 h, and HEK293T cells were stimulated with TNFα (10 ng/ml) for 8 h. Reporter activity was analyzed using the dual luciferase assay kit (Promega).

### Open field test

The open field test (OFT) was used to assess spontaneous activity, anxiety-like behavior, and emotional change in the animals (*n* = 12 per group for each experiment), mice were placed in the corner of a plastic box (40 cm × 40 cm × 40 cm) and moving freely, the base of the box was divided into 16 equal sectors, the time spent in each area, horizontal and vertical activity, frequency of urination, and defecation were monitored for 5 min. The open field box was cleaned with 75% ethyl alcohol after each test.

### Morris water maze test

The Morris water maze studies were performed to measure spatial learning memory and cognitive flexibility function of the mice (*n* = 12 per group for each experiment). The round tank was 120 cm in diameter, 30 cm in depth with several visual clues around, and filled with water (25 °C) with white non-toxic paint. The maze was divided into four quadrants; a submerged platform 1 cm below the surface was placed at a fixed location and maintained in the same position during all trials. All mice receive five consecutive days training with four spatial acquisition trials to find the hidden platform within 60 s each day. If the mice fail to find the platform within the time limit, the mice were gently guided to the platform and stayed for 10 s. On day 6, a probe trial was conducted with the platform removed. A video camera above was used for the record of swimming speed, latency to escape to the platform, the percentage of time spent in the target quadrant, and distance swam to the platform.

### Y-maze test

Y-maze (*n* = 12 per group for each experiment) consists of a three-armed chamber, with the arms at a 120° angle from each other. Each arm is 35 cm long, 5.0 cm wide, and 10 cm high. Y-maze testing was conducted as reported previously. The mice were put into the neutral zone of the Y-maze, and arm entries were recorded for 5 min. Alternation behavior was defined as consecutive entries into all three arms without repeated entries and was expressed as a percentage of the total arm entries.

### Statistical analysis

**Fig. 1:**
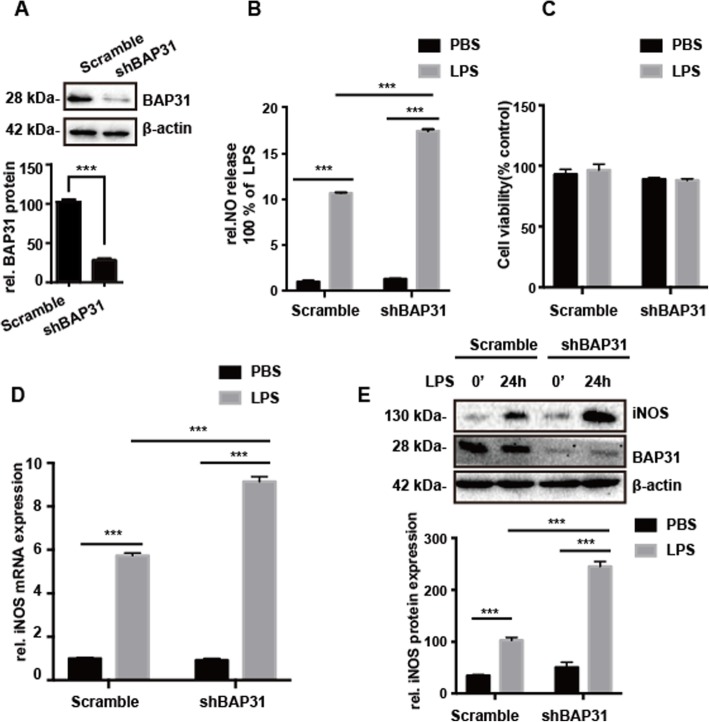
BAP31-deficient BV2 cells produce more NO upon LPS challenge. **a** Immunoblot analysis of BAP31 in lysates of scramble and shBAP31 BV2 cells. **b** NO production was measured by Griess assay. **c** Cell viability was measured by MTT assay. **d** Quantitative RT-PCR measured the relative mRNA expression of iNOS in LPS-treated scramble and shBAP31 BV2 cells. **e** Immunoblot analysis of iNOS in scramble and shBAP31 BV2 cells stimulated with LPS for 24 h. All the data are indicated as Mean ± SEM of three independent experiments. *P < 0.05; **P < 0.01; ***P < 0.001 versus control group

Statistical analyses were conducted with GraphPad Prism 7.0 Software (GraphPad, La Jolla, CA, USA). Data are expressed as the mean ± SEM. Data presented in Figs. 1a, 4c, d, and 7c–e were analyzed by using Student’s *t* test between two groups. Data presented in Fig. 5d, e were analyzed by using one-way ANOVA followed by a Tukey-Kramer multiple comparison post hoc test. Two-way ANOVA followed by a Tukey-Kramer multiple comparison post hoc test was used for comparisons of three or more groups in all other experiment results. *P < 0.05, **P < 0.01, ***P < 0.001, and n.s. (no significant difference) denote the significance thresholds.

## Results

### BAP31 deficiency exacerbates LPS-induced NO release by increasing iNOS expression

First, we employed an shRNA approach to specifically knockdown BAP31 in microglial BV2 cells. Using immunoblotting, the protein level of BAP31 was reduced by 70% compared with that of scrambled shRNA cells (Fig. 1a). The effect of BAP31 on microglial activation was confirmed using nitrite assays. Nitric oxide (NO) production in LPS-treated scramble BV2 cells increased to 10.70 ± 0.08-fold compared to that of scramble BV2 cells, but with BAP31 protein knockdown; NO formation increased to 17.52 ± 0.17-fold, without affecting cell viability (Fig. 1b, c). Inducible nitric oxide synthase (iNOS), which produces a large amount of NO, is induced in microglia in response to inflammatory mediators such as LPS and cytokines [21]. Therefore, we assessed whether BAP31 affects NO release by iNOS production. We measured the mRNA and protein levels of iNOS in LPS-stimulated BV2 cells, and found that BAP31 deficiency significantly increased the mRNA (from 5.80 ± 0.01-fold to 8.30 ± 0.18-fold) and protein (from 102.65 ± 4.72% to 244.92 ± 7.96%) expression levels of iNOS versus those of the scramble cells, indicating that BAP31 deficiency enhanced LPS induced NO release by increasing iNOS expression (Fig. 1d, e).

### BAP31 deficiency exacerbates the production of inflammatory cytokines induced by LPS

**Fig. 2:**
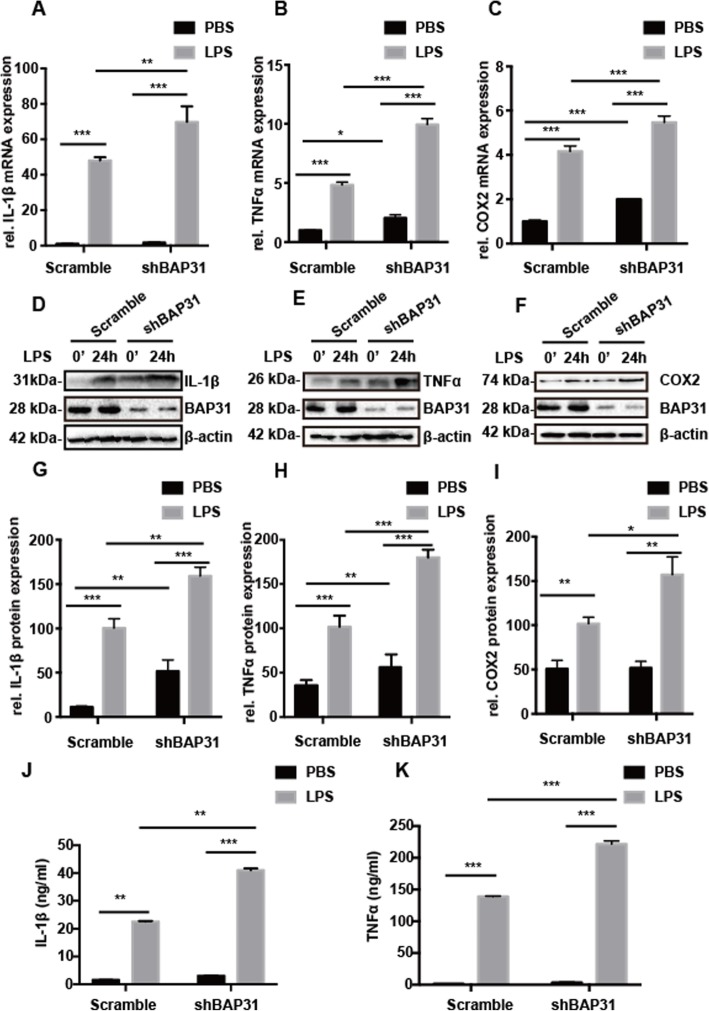
BAP31-deficient BV2 cells produce more inflammatory cytokines. Scramble and shBAP31 were treated with LPS (100 ng/ml) for 4 h. The mRNA levels of the cytokines IL-1β (**a**), TNFα (**b**), and COX2 (**c**) were analyzed with RT-PCR. Scramble and shBAP31 were treated with LPS (100 ng/ml) for 24 h. The protein levels of the cytokines IL-1β (**d**, **g**), TNFα (**e**, **h**), and COX2 (**f**, **i**) in cells were analyzed with Western bloting; the secreted protein levels of the cytokines IL-1β (**j**) and TNFα (**k**) in supernatant were analyzed with ELISA kits. All the data are indicated as Mean ± SEM of three independent experiments. *P < 0.05; **P < 0.01; ***P < 0.001 versus control group

To confirm the influence of BAP31 on the inflammatory response, we assessed the impact of BAP31 on proinflammatory cytokines in response to LPS. The levels of the proinflammatory cytokines IL-1β, TNFα, and COX2 were measured in BV2 cells. BAP31 deficiency elevated the mRNA expression of IL-1β from 1.00 ± 0.02-fold to 1.73 ± 0.10-fold, TNFα from 1.00 ± 0.01-fold to 2.04 ± 0.15-fold, and COX2 from 1.01 ± 0.08-fold to 1.91 ± 0.16-fold. Exposure to LPS significantly increased the mRNA production of IL-1β, TNFα, and COX2 in both scramble and shBAP31 cells, but BAP31 deficiency exacerbated the cytokine mRNA production, increasing IL-1β from 49.16 ± 0.20-fold to 69.86 ± 5.01-fold, TNFα from 4.97 ± 0.05-fold to 9.94 ± 0.29-fold, and COX2 from 4.10 ± 0.20-fold to 5.51 ± 0.23-fold (Fig. 2a–c, respectively). In addition, we assessed the protein expression of IL-1β, TNFα, and COX2 after LPS administration for 24 h, and the results were consistent with the mRNA production. The protein levels of IL-1β, TNFα, and COX2 significantly increased after LPS administration in both groups, but BAP31-deficient cells increased IL-1β from 100.30 ± 2.86% to 159.11 ± 1.41% (Fig. 2d, g), TNFα from 100.00 ± 2.83% to 180.10 ± 5.02% (Fig. 2e, h), and COX2 from 101.70 ± 4.41% to 157.31 ± 7.79% in scramble and shBAP31 cells (Fig. 2f, i), respectively. Secreted IL-1β and TNFα protein levels were detected by ELISA assay after treatment with LPS for 24 h, and consistent with the results above, the levels of secreted IL-1β and TNFα significantly increased after LPS administration in both groups, but BAP31 deficiency exacerbated the secretion of IL-1β and TNFα, increasing IL-1β from 22.61 ± 0.19 pg/ml to 41.29 ± 0.71 pg/ml (Fig. 2j) and TNFα from 138.90 ± 0.94 pg/ml to 221.60 ± 2.89 pg/ml (Fig. 2k).

### BAP31 deficiency accelerates the translocation and transcriptional activity of NF-κB p65 and c-Jun

**Fig. 3:**
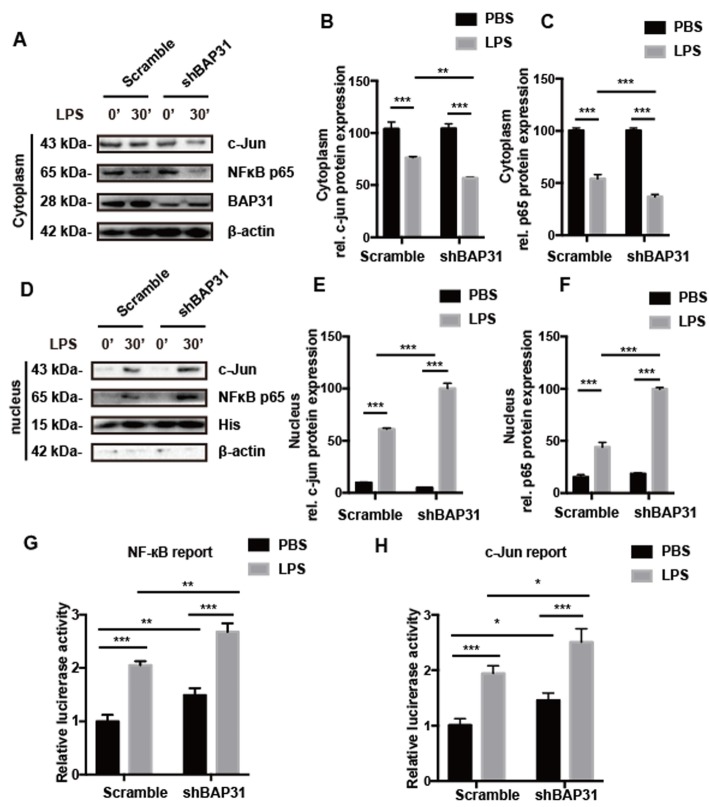
BAP31 deficiency facilitates p65 and c-Jun nuclear accumulation and transcriptional activity. Scramble and shBAP31 BV2 cells were treated with LPS for 30 min; the cytosolic (**a**) and nuclear (**d**) fractions of p65 and c-Jun were analyzed by Western blotting with antibodies to c-Jun, p65, histone, and β-actin. Immunoblots for c-Jun (**b**) and p65 (**c**) in cytosolic fractions were quantified and normalized to β-actin protein. Immunoblots for c-Jun (**e**) and p65 (**f**) in nuclear fractions were quantified and normalized to histone protein. **g**, **h** Scramble and shBAP31 Raw264.7 cells were cotransfected with NF-κB luciferase reporter plasmid or AP-1 luciferase reporter plasmid and pRL-SV40-C plasmid for 48 h, and p65 and c-Jun reporter activities were analyzed following treatment with LPS for 24 h. All the data are indicated as Mean ± SEM of three independent experiments. *P < 0.05; **P < 0.01; ***P < 0.001 versus control group

The previous study found that pro-inflammatory cytokine production and secretion are regulated by the proteins of the MAPK and NF-κB pathways [22, 23]. Based on the NO release and cytokine production results, we tested whether BAP31 deficiency affects cytokine production under the control of NF-κB p65 and MAPK-dependent AP-1 transcription factors. Scramble and shBAP31 cells were exposed to LPS for 30 min, and then the nuclei were separated from the cytoplasm. As shown in Fig. 3a–f, upon LPS stimulation, the translocation of NF-κB p65 and c-Jun from the cytoplasm to the nucleus significantly increased, but BAP31-deficient cells had increased translocation of NF-κB p65 and c-Jun proteins compared with that of scramble cells. In scramble cells, the protein level of NF-κB p65 decreased from 100.31 ± 1.45% to 54.13 ± 2.29% and the protein level of c-Jun decreased from 103.91 ± 3.87% to 82.37 ± 0.71% in the cytoplasm, while in shBAP31 cells, the protein level of NF-κB p65 decreased from 100.53 ± 1.35% to 36.75 ± 1.37% and the protein level of c-Jun decreased from 104.21 ± 2.61% to 60.84 ± 0.99% in the cytoplasm; accordingly, in scramble cells, the protein level of NF-κB p65 increased from 15.36 ± 1.28% to 43.95 ± 2.74% and the protein level of c-Jun increased from 9.74 ± 0.62% to 67.93 ± 1.11% in the nucleus, while in shBAP31 cells, the protein level of NF-κB p65 increased from 18.52 ± 0.93% to 100.36 ± 3.60% and the protein level of c-Jun increased from 5.02 ± 0.38% to 60.84 ± 0.99% in the nucleus.

Furthermore, we detected the NF-κB p65 and c-Jun transcriptional activity in Raw264.7 scramble and shBAP31 cells, the protein level of BAP31 was reduced by 70% compared with that of scrambled shRNA cells (Additional file 1). As shown in Fig. 3g, h, BAP31 deficiency enhanced the transcriptional activity following LPS stimulation, increasing NF-κB p65 from 2.05 ± 0.07% to 2.68 ± 0.16% and c-Jun from 1.94 ± 0.14% to 2.51 ± 0.24%, and this transcription activity was significantly increased compared with that of the scramble cells. To verify these results, we detected the NF-κB p65 and c-Jun transcriptional activity in HEK293T cells, and when stimulated with TNFα, NF-κB p65 (from 1.32 ± 0.11% to 1.77 ± 0.15%) and c-Jun (from 1.54 ± 0.14% to 1.94 ± 0.13%) transcriptional activity was significantly increased compared with that of the scramble cells (Additional file 2), consistent with the results of the from Raw264.7 cells. These results suggest that BAP31 deficiency increases NO release and cytokine production by enhancing the translocation and transcriptional activity of NF-κB p65 and c-Jun.

### BAP31 regulates the translocation of NF-κB p65 and c-Jun through IRAK1

**Fig. 4:**
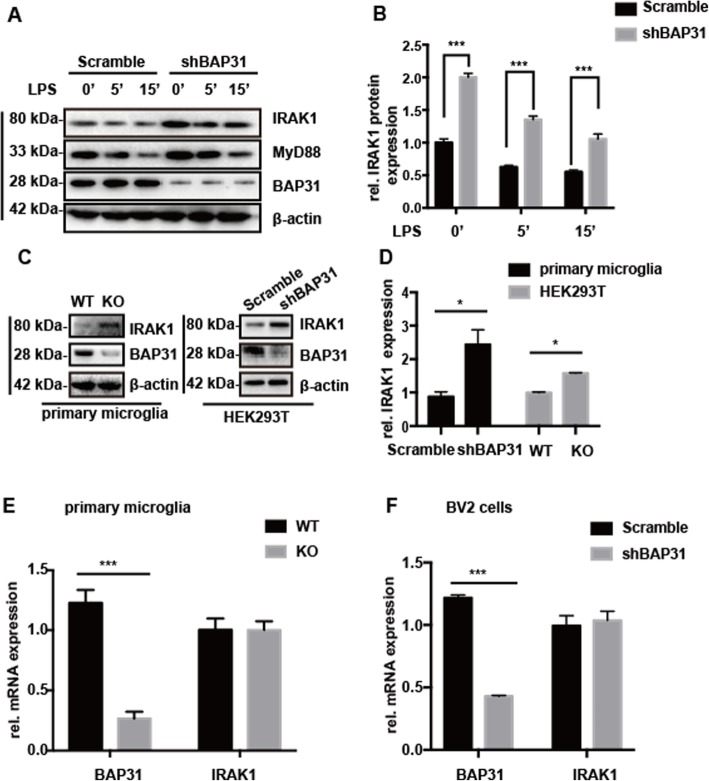
Effect of BAP31 deficiency on the protein levels of IRAK1 and MyD88 in BV2 cells. **a**, **b** Scramble and shBAP31 BV2 cells were treated with LPS for 0, 5, and 15 min. Representative Western blots showing the expression levels of IRAK1 and MyD88. **c**, **d** Representative Western blots showing the effect of BAP31 on the protein level of IRAK1 in primary microglia and HEK293T cells. **e** Quantitative RT-PCR measured the relative mRNA expression of IRAK1 in primary WT and KO microglial cells. **f** Quantitative RT-PCR measured the relative mRNA expression of IRAK1 in scramble and shBAP31 BV2 cells. All the data are indicated as Mean ± SEM of three independent experiments. *P < 0.05; **P < 0.01; ***P < 0.001 versus control group

We continued to investigate the mechanism by which BAP31 promots the translocation and transcriptional activity of NF-κB p65 and c-Jun. We found that BAP31 deficiency had no effect on the mRNA production of TLR4 and MyD88 (Additional file 3). To investigate whether BAP31 deficiency interferes with the protein level and LPS-induced degradation of MyD88, cells were stimulated with LPS for 5 min and 15 min to induce MyD88 degradation. As shown in Fig. 4a, BAP31 deficiency had no significant effect on the protein level and LPS-induced time-dependent degradation of MyD88 (Fig. 4a and Additional file 4), suggesting that BAP31 knockdown-mediating inflammation does not involve MyD88. However, we found that BAP31 deficiency significantly upregulated the protein level of IRAK1 in BV2 cells, from 1.00 ± 0.057-fold to 2.02 ± 0.59-fold (Fig. 4a). This result was verified in primary microglial cells (from 0.96 ± 0.24-fold to 2.43 ± 0.77-fold) and HEK293T cells (from 0.99 ± 0.22-fold to 1.58 ± 0.02-fold) (Fig. 4c, d). LPS also induced time-dependent degradation of IRAK1 (Fig. 4a, b), but IRAK1 remained at a high level in BAP31-deficient cells, highlighting BAP31 as an important mediator of IRAK1. Then, we detected whether the mRNA level of IRAK1 was influenced by BAP31, as and illustrated in Fig. 4e, f, BAP31 konckdown (Additional file 5 and 6) induced no significant change in the mRNA level of IRAK1 in primary microglia and BV2 cells, indicating that BAP31 deficiency increased the protein level of IRAK1 might through posttranscriptional mechanisms.

### Silencing IRAK1 alleviates the production of inflammatory factors caused by BAP31 deficiency

**Fig. 5:**
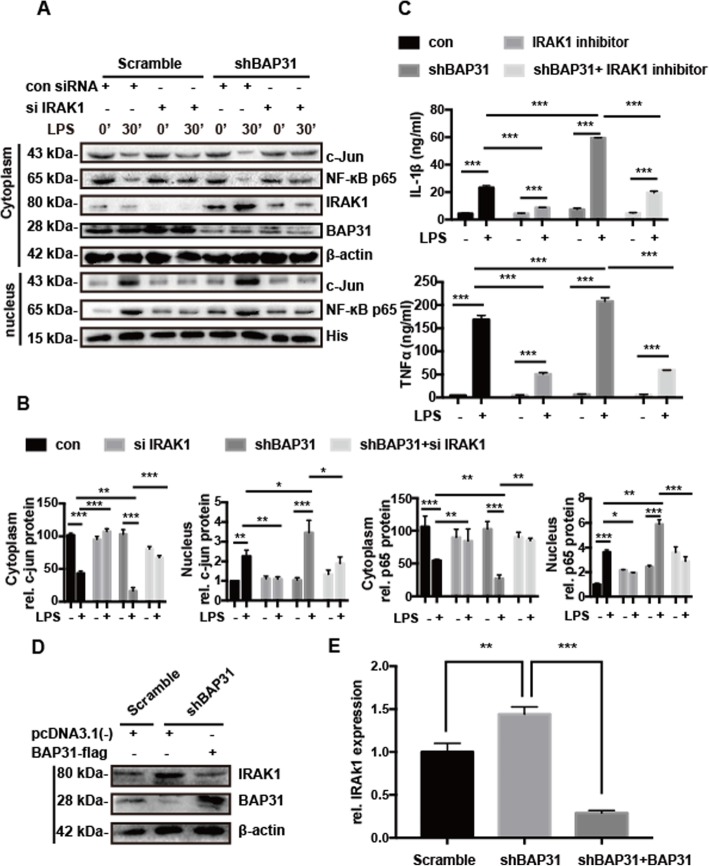
Silencing IRAK1 alleviates the production of inflammatory factors caused by BAP31 deficiency. **a** Scramble and shBAP31 BV2 cells were transfected with IRAK1 siRNA for 60 h, followed by treatment with LPS for 30 min. The cytosolic and nuclear fractions were analyzed by Western blotting with antibodies to c-Jun, p65, histone, and β-actin. **b** Immunoblots for c-Jun and p65 in cytosolic fractions were quantified and normalized to β-actin protein; immunoblots for c-Jun and p65 in nuclear fractions were quantified and normalized to histone protein. **c** Scramble and shBAP31 BV2 cells were treated with IRAK1 inhibitor for 48 h, followed by stimulation with LPS for 24 h. The secreted protein levels of the cytokines IL-1β and TNFα in supernatant were analyzed with ELISA kits. **d**, **e** Scramble and shBAP31 HEK293T cells were transfected with pcDNA3.1(−) and BAP31-flag plasmids, respectively, for 48 h. Cell lysates were analyzed by Western blotting with antibodies to IRAK1, BAP31, and β-actin. All the data are indicated as Mean ± SEM of three independent experiments. *P < 0.05; **P < 0.01; ***P < 0.001 versus control group

To detect whether BAP31 deficiency increases cytokine production through IRAK1, scramble and shBAP31 BV2 cells were transfected with IRAK1 siRNA for 60 h. The cells were exposed to LPS for 30 min, and then the nuclei were separated from the cytoplasm. As shown in Fig. 5a, b, IRAK1 deficiency significantly inhibited the translocation of NF-κB p65 and c-Jun from the cytoplasm to the nucleus in scramble and shBAP31 BV2 cells. The amount of NF-κB p65 in the cytoplasm decreased to 29.91 ± 3.28% in shBAP31 BV2 cells versus to 84.99 ± 2.19% in IRAK1-deficiency shBAP31 BV2 cells. The amount of c-Jun in the cytoplasm decreased to 16.35 ± 2.98% in shBAP31 BV2 cells versus to 67.45 ± 1.83% in IRAK1-deficiency shBAP31 BV2 cells. Accordingly, the nucleus translocation of NF-κB p65 (from 5.90 ± 0.21-fold to 2.88 ± 0.21-fold) and c-Jun (from 3.45 ± 0.37-fold to 1.89 ± 0.19-fold) decreased in IRAK1-deficiency shBAP31 BV2 cells. To further verify that BAP31 deficiency increases cytokine production through IRAK1, we detected the translocation of NF-κB p65 and c-Jun in scramble and shBAP31 BV2 cells when treated with an IRAK1 inhibitor for 48 h. The cells were exposed to LPS for 30 min, and then the nuclei was separated from the cytoplasm. As shown in Additional file 7, the translocation of NF-κB p65 and c-Jun from the cytoplasm to nucleus was significantly inhibited in the scramble and shBAP31 BV2 cells. The amount of NF-κB p65 in the cytoplasm decreased to 32.93 ± 2.90% in shBAP31 BV2 cells versus to 86.27 ± 2.76% in IRAK1 inhibitor-treated shBAP31 BV2 cells. The amount of c-Jun in the cytoplasm decreased to 34.85 ± 2.38% in shBAP31 BV2 cells versus to 70.52 ± 6.98% in IRAK1 inhibitor treated shBAP31 BV2 cells. Accordingly, nuclear translocation of NF-κB p65 (from 100.07 ± 5.78% to 69.56 ± 1.31%) and c-Jun (from 100.15 ± 5.77% to 66.18 ± 2.13%) decreased in IRAK1 inhibitor treated shBAP31 BV2 cells. Then we tested whether inhibiting IRAK1 could affect the secretion of inflammatory factors induced by BAP31 deficiency. The scramble and shBAP31 cells were pretreated with an IRAK1 inhibitor for 48 h and then stimulated with LPS for 24 h, and the supernatants were analyzed by ELISA. The secreted protein levels of IL-1β and TNFα in both groups significantly decreased when incubated with the IRAK1 inhibitor, and as illustrated in Fig. 5c, IL-1β decreased from 23.24 ± 0.86 pg/ml to 8.65 ± 0.14 pg/ml, and TNFα decreased from 168.61 ± 5.24 pg/ml to 50.54 ± 1.95 pg/ml in scramble cells; IL-1β decreased from 59.46 ± 0.14 pg/ml to 19.59 ± 1.08 pg/ml, and TNFα decreased from 207.70 ± 4.47 pg/ml to 59.46 ± 0.14 pg/ml in shBAP31 cells. These results suggest that BAP31 might influence the inflammatory response through IRAK1.

Since the knockdown of BAP31 upregulated the expression level of IRAK1, we subsequently examined whether recovering the protein expression of BAP31 revert the level of IRAK1 to normal. Hence, the BAP31-flag plasmid was used to overexpress BAP31 in shBAP31 HEK293T cells, and as illustrated in Fig. 5d, e, when BAP31 protein was overexpressed, the protein levels of IRAK1 reverted from 1.44 ± 0.05-fold to 0.29 ± 0.02-fold. Thus, these results confirm that BAP31 regulates IRAK1 expression.

### BAP31 deficiency enhances the activation of NF-κB and MAPK pathways in BV2 cells

**Fig. 6:**
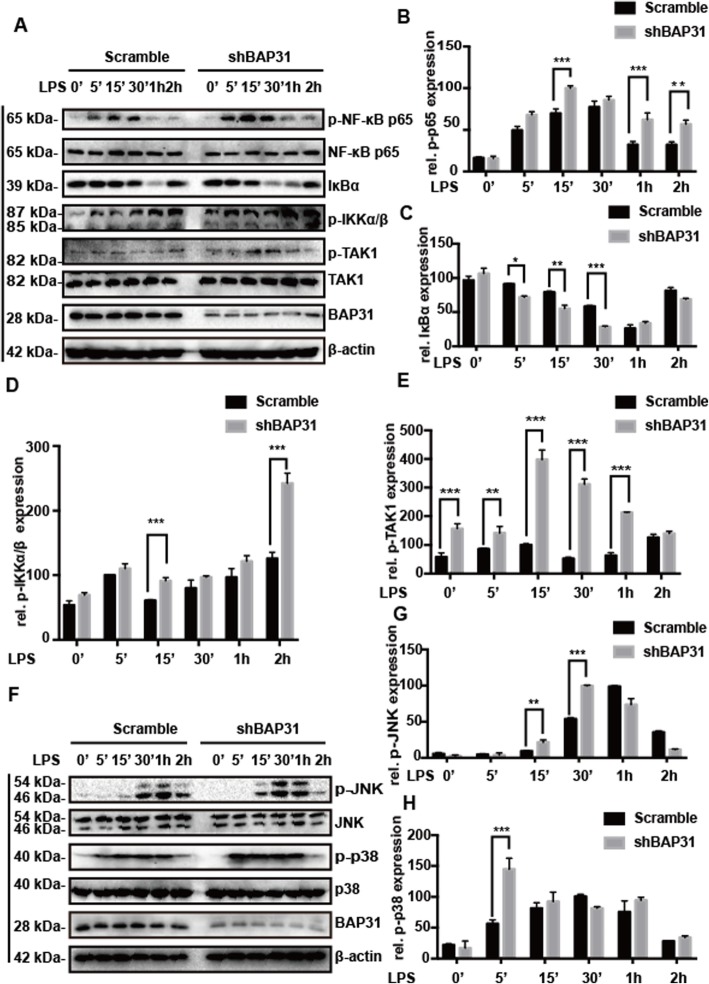
Effect of BAP31 deficiency on the nuclear factor-κB and mitogen-activated protein kinase (MAPK) pathways in BV2 cells. Scramble and shBAP31 BV2 cells were treated with LPS for 0 min, 5 min, 15 min, 30 min, 1 h, 2 h. **a** Representative Western blots showing the expression levels of total and phospho-p65, IκBα, phospho-IKKα/β, and total and phospho-TAK1. The intensity of the phospho-p65 (**b**) and phospho-TAK1 (**e**) protein bands were quantified and shown as the ratio of phosphorylated protein/total protein to control after normalization to β-actin. The intensity of the IκBα (**c**) and phospho-IKKα/β (**d**) proteins bands were quantified and shown as the ratio to control after normalization to β-actin. **f** Representative Western blots showing expression levels of total and phospho-JNK, total and phospho-p38. The intensity of the phospho-JNK (**g**) and phospho-p38 (**h**) protein bands were quantified and shown as the ratio of phosphorylated protein/total protein to control after normalization to β-actin. All the data are indicated as Mean ± SEM of three independent experiments. *P < 0.05; **P < 0.01; ***P < 0.001 versus control group

IRAK1 is an adaptor proteins that functions in a signaling hub that connects various immune receptors to the downstream signaling cascades, such as the Jun amino-terminal kinase, p38 mitogen-activated protein kinases (MAPK), extracellular signal-regulated kinase, and NF-κB activation pathways [24–28]. NF-κB and MAPK signaling pathways play critical roles in immunoinflammatory reactions, and we examined the impact of BAP31 knockdown on LPS-mediated signaling downstream of IRAK1. To determine whether BAP31 influences these signaling pathways after LPS stimulation, we analyzed the phosphorylation of TAK1, IKKα/β, NF-κB p65, and MAPKs [p38MAPK and Jun N-terminal kinase 1/2 (JNK)] and the degradation of IĸBα in BV2 cells treated with LPS for 5 min, 15 min, 30 min, 1 h, and 2 h. We found that TAK1 activation greatly increased in BAP31-deficient cells upon LPS stimulation compared with that of the scramble cells. Active TAK1 significantly promoted the phosphorylation of IKKα/β, p38 and JNK, and IKK activation led to the degradation of IκBα, which promoted the phosphorylation and translocation of NF-κB p65 (Fig. 6a, f). The phosphorylation level of TAK1 increased from 104.21 ± 3.94% to 435.54 ± 7.35% at 15 min (Fig. 6e). The phosphorylation level of IKKα/β increased from 59.61 ± 1.10% to 94.34 ± 5.04% at 15 min (Fig. 6d). IκBα was degraded upon LPS stimulation, and knockdown of BAP31 promoted LPS-induced IĸBα degradation from 58.25 ± 1.22% to 27.86 ± 1.07% at 30 min (Fig. 6c). The phosphorylation level of NF-κB p65 increased from 69.77 ± 5.26% to 100 ± 2.89% at 15 min (Fig. 6b). The phosphorylation level of p38 increased from 52.42 ± 1.22% to 139.02 ± 1.07% (Fig. 6h), and the phosphorylation level of JNK increased from 52.26 ± 1.65% to 98.35 ± 1.19% (Fig. 6g), suggesting a critical role of BAP31 in the NF- κB p65 and MAPK activation pathways.

### Conditional BAP31 knockout mice exhibit more inflammation when administered LPS

**Fig. 7:**
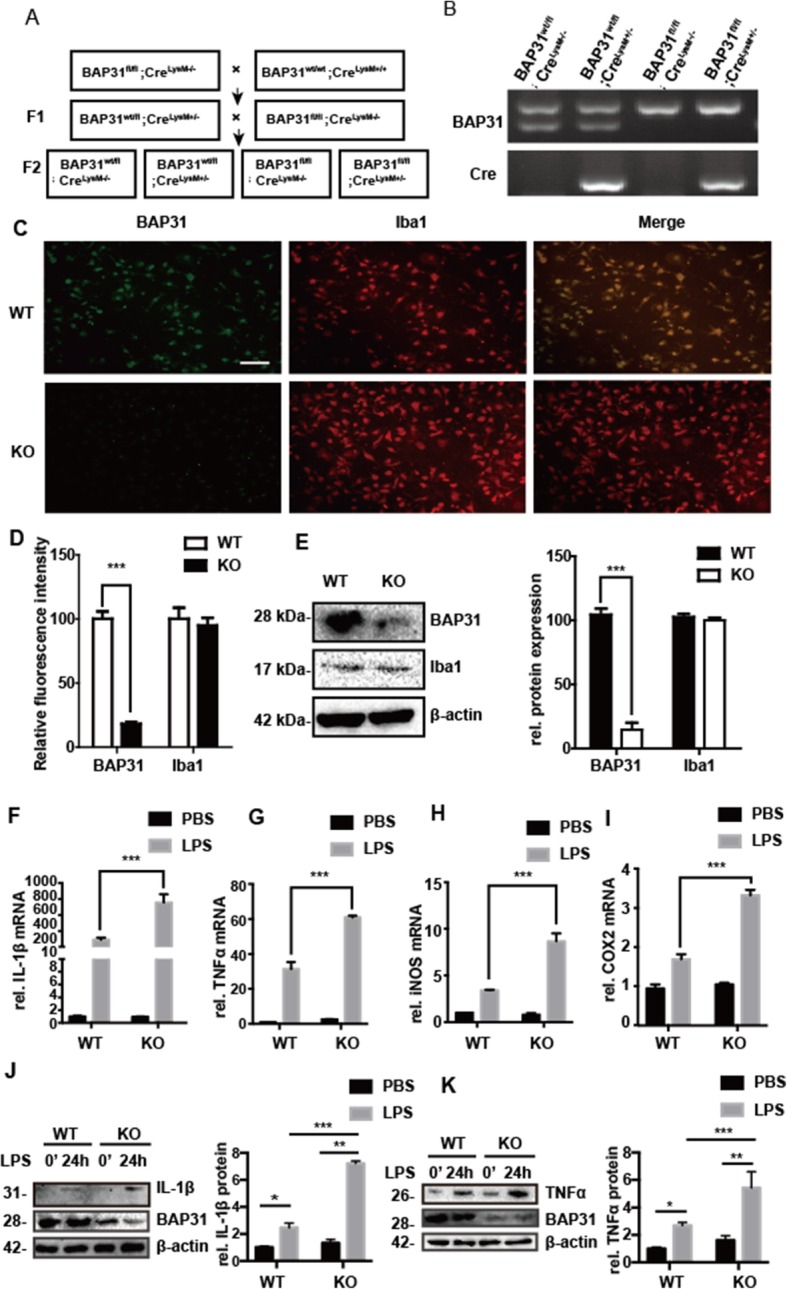
Conditional microglia BAP31 knockout mice exhibit more inflammation when administered LPS. **a** Schematic representation of the breeding strategy. The representation of the breeding strategy. **b** PCR analysis of BAP31^fl/fl^, BAP31^wt/wt^, and Cre genes in the second offspring. **c** Immunocytochemistry for BAP31 (green) and Iba1 (red) was performed in the primary microglial cells of the WT and KO mice. Scale bar = 50 μm. **d** The relative immunofluorescence intensity was used to represent the protein levels of BAP31 and Iba1. **e** Western blot analysis of BAP31and Iba1 in primary microglia of the WT and KO mice. **f**–**i** Levels of IL-1β, TNFα, iNOS, and COX2 mRNA in samples of the hippocampus were analyzed by RT-PCR. Primary WT and KO microglial cells were treated with LPS (100 ng/ml) for 24 h. The protein levels of the cytokines IL-1β (**j**) and TNFα (**k**) in cells were analyzed by Western bloting; all the data are indicated as Mean ± SEM of three independent experiments. *P < 0.05; **P < 0.01; ***P < 0.001 versus control group

Microglia are unique glial cells derived from common myeloid progenitors during the developmental stages of the CNS. To explore the physiological role of BAP31, we generated BAP31-deficient mice. Cre ^LysM-/-^ BAP31^fl/fl^ and Cre ^LysM+/-^ BAP31^fl/fl^ mice were crossed to target BAP31 in microglia, and the offspring were 50% Cre ^LysM+/-^ BAP31^fl/fl^ and 50% Cre ^LysM-/-^ BAP31^fl/fl^. We denoted Cre ^LysM-/-^ BAP31^fl/fl^ as wild-type (WT) and Cre ^LysM+/-^ BAP31^fl/fl^ as Knockout (KO). The specific breeding strategy is shown in the Fig. 7a. The insertion of the targeted allele flanked with LoxP sites and the deleted allele after Cre recombination is illustrated in the Additional file 8. To verify the deletion of BAP31 in the microglia, the gene knockdown was first confirmed by genotyping, as shown in Fig. 7b. The absence of microglial BAP31 expression in the hippocampus of Cre ^LysM-/-^ BAP31^fl/fl^ and Cre ^LysM+/-^ BAP31^fl/fl^ mice was performed using dual BAP31 and Iba1 (green, anti-BAP31; red, anti-Iba1) immunofluorescence analysis. The distribution of immunofluorescence intensity indicated that BAP31 was not expressed in microglia (Additional file 9). In primary microglial cells (green, anti-BAP31; red, anti-Iba1) isolated from the mouse model, the expression of BAP31 was depleted in Cre ^LysM+/-^ BAP31^fl/fl^ mice, as shown in the quantitative data (Fig. 7c, d). From the Western blot analysis, we also confirmed that the BAP31 expression was depleted in microglia (Fig. 7e).

To verify the function of BAP31 in microglia in vivo, we examined the hippocampus of the mice described above for inflammation by assessing the expression of the proinflammatory cytokines, IL-1β, TNFα, iNOS, and COX2 by RT-PCR. Cre ^LysM+/-^ BAP31^fl/fl^ mice administered LPS had a higher expression (Fig. 6f–i) of all four cytokines compared with that of the Cre ^LysM-/-^ BAP31^fl/fl^ mice (IL-1β: 754.87 ± 8.54-fold versus 290.04 ± 3.28-fold; TNFα: 60.97 ± 2.69-fold versus 31.34 ± 3.41-fold; iNOS: 3.41 ± 0.034-fold versus 8.66 ± 0.50-fold; COX2: 8.66 ± 1.71-fold versus 3.41 ± 2.48-fold respectively). Consistent with the in vitro results, BAP31 deficiency significantly exacerbated the cytokine production.

In addition, we assessed the protein expression of IL-1β and TNFα in primary microglial cells after LPS administration for 24 h, and consistent with the mRNA results in the hippocampus, the protein levels of IL-1β and TNFα significantly increased after LPS administration in both groups, but cells from KO mice produced more IL-1β (from 2.47 ± 0.19-fold to 7.21 ± 0.18-fold) (Fig. 7j) and TNFα (from 2.69 ± 0.13-fold to 5.43 ± 0.68-fold) (Fig. 7k) than WT microglial cells.

**Fig. 8:**
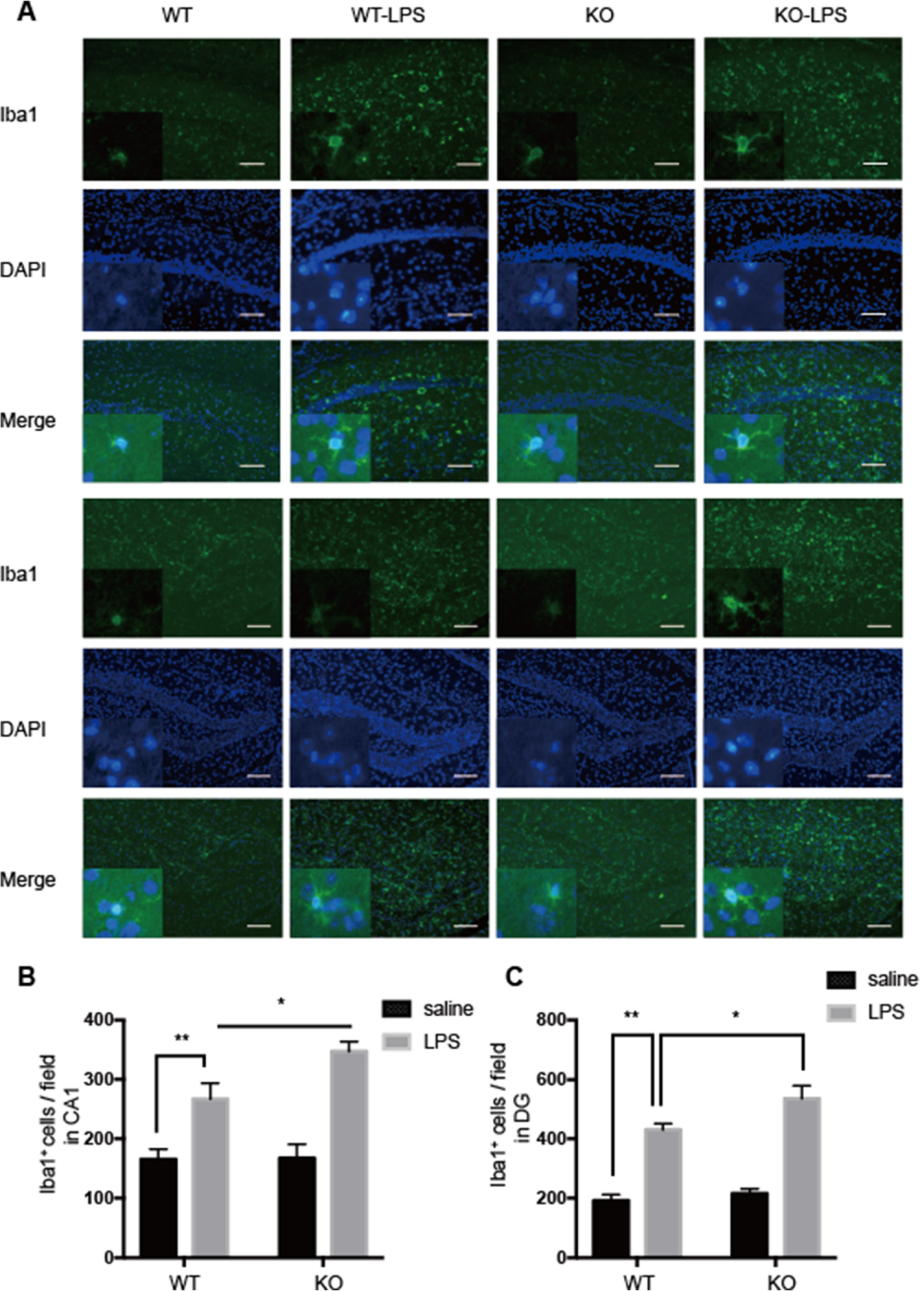
BAP31 attenuates lipopolysaccharide (LPS)-induced microglial activation. **a** Representative images of Iba1-labeled activated microglia in the hippocampal CA1 and DG areas. Activated microglia are shown in green. Scale bars = 200 μm. **b** Density of Iba1^+^ cells in the DG. **c** Density of Iba1^+^ cells in the DG. *n* = 12 per group for each experiment. Data are expressed as mean ± SEM. *P < 0.05; **P < 0.01; ***P < 0.001 versus control group

Ionized calcium binding adapter molecule 1 (Iba1): a calcium-binding protein expressed exclusively by microglia in the central nervous system [29], is a measure of microglial reactivity following insult to the CNS, and when microglial cells are activated, the protein expression of Iba1 increases [30, 31]. Since the hippocampus is an area of the brain that is responsible for learning and memory and exhibits cognitive impairment in patients with neurodegenerative disease [32], we next assessed the Iba1 expression in the hippocampus of these mice after LPS or saline administration. The average number of Iba1-positive cells per field of view was significantly increased in Cre ^LysM-/-^ BAP31^fl/fl^ mice following LPS administration compared with that of Cre ^LysM-/-^ BAP31^fl/fl^ mice following saline administration, as indicated in Fig. 8a, but LPS-treated Cre ^LysM+/-^ BAP31^fl/fl^ mice showed more Iba1 expression compared with that of LPS-treated Cre ^LysM-/-^ BAP31^fl/fl^ mice in the CA1 region (the upper three lines; Cre ^LysM-/-^ BAP31^fl/fl^+ LPS 267.10 ± 26.56 versus Cre ^LysM+/-^ BAP31^fl/fl^ + LPS 347.10 ± 16.23; Fig. 8b) and DG region (the bottom three lines; Cre ^LysM-/-^ BAP31^fl/fl^+ LPS 424.30 ± 20.43 versus Cre ^LysM+/-^ BAP31^fl/fl^ + LPS 536.60 ± 43.22; Fig. 8c), which had a ramified morphology with enhanced activity, indicating that BAP31 might stabilize the microglial activation in vivo.

**Fig. 9:**
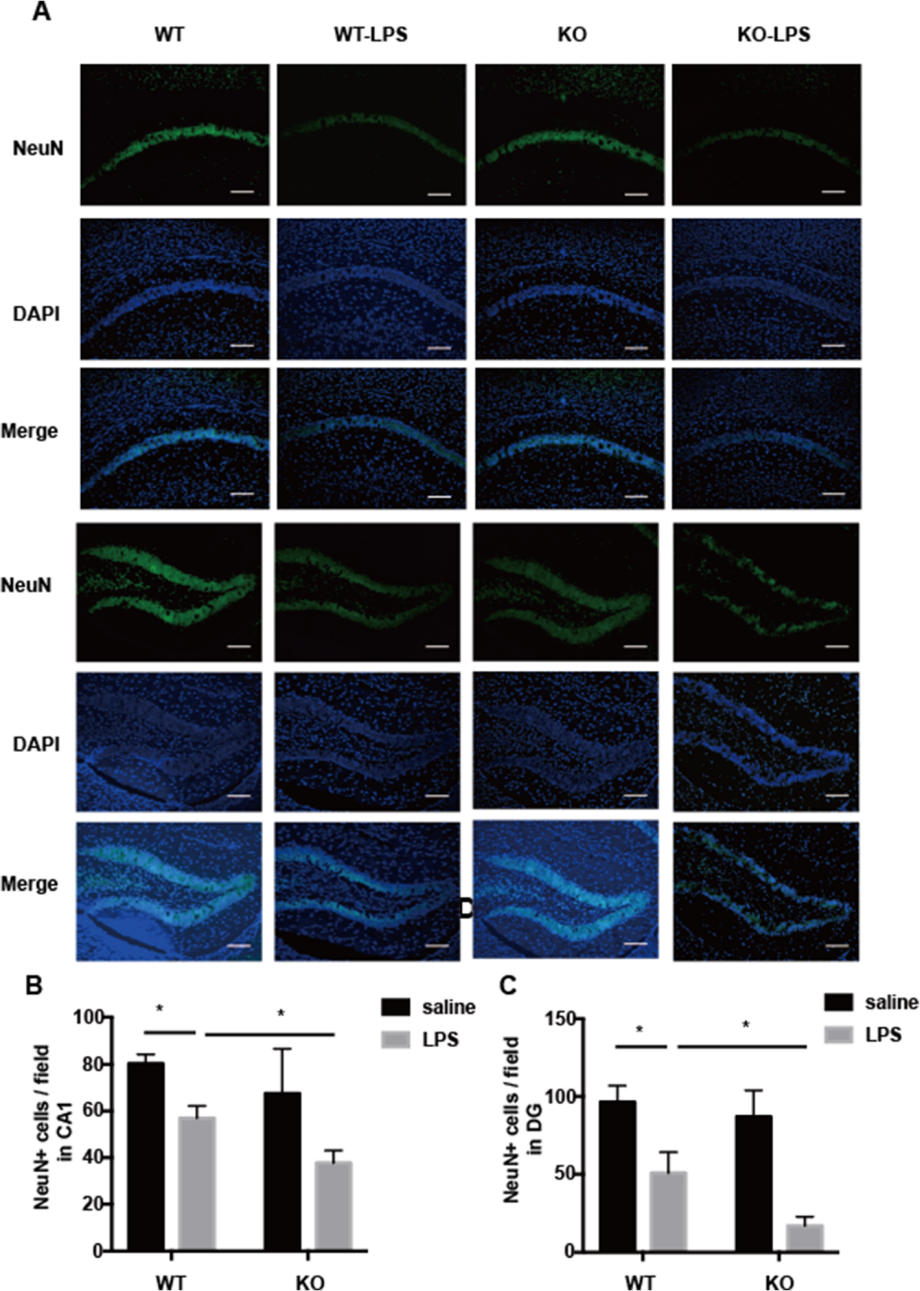
BAP31 protects neurons from lipopolysaccharide (LPS)-induced inflammatory cytokines. **a** Representative images of NeuN-labeled intact neurons in the hippocampal CA1 and DG areas. The intact neuron is shown in green. Scale bars = 200 μm. **b** Density of NeuN^+^ cells in the DG. (c) Density of NeuN^+^ cells in the DG. *n* = 12 per group for each experiment. *n* = 12 per group for each experiment. Data are expressed as mean ± SEM. *P < 0.05; **P < 0.01; ***P < 0.001 versus control group.

As neuroinflammation is known to damage neurons, we then determined neuronal integrity using a NeuN antibody, which detects intact neurons. Similar sections of the hippocampus were compared between the four groups of mice. In the Cre ^LysM-/-^ BAP31^fl/fl^ groups, NeuN-positive cells were densely packed in the DG and CA1 regions, after LPS challenge, and the total numbers of NeuN-positive cells per field in the DG and CA1 regions were significantly decreased following treatment with LPS, as indicated in Fig. 9a, but BAP31 deficiency significantly decreased the number of NeuN-positive cells compared with that of the Cre ^LysM-/-^ BAP31^fl/fl^ + LPS group in CA1 region (the upper three lines; Cre ^LysM-/-^ BAP31^fl/fl^ + LPS group 56.98 ± 5.16 versus Cre ^LysM+/-^ BAP31^fl/fl^ + LPS 37.74 ± 5.29; Fig. 9b) and DG region (the bottom three lines; Cre ^LysM-/-^ BAP31^fl/fl^ + LPS 50.85 ± 13.29 versus Cre ^LysM+/-^ BAP31^fl/fl^ + LPS 17.07 ± 5.81; Fig. 9c). Thus, we determined that BAP31 deficiency exacerbates neuronal death by exacerbating the inflammatory response after LPS administration in vivo.

### BAP31 deficiency exacerbates memory deficits caused by experimental cerebral inflammation

**Fig. 10:**
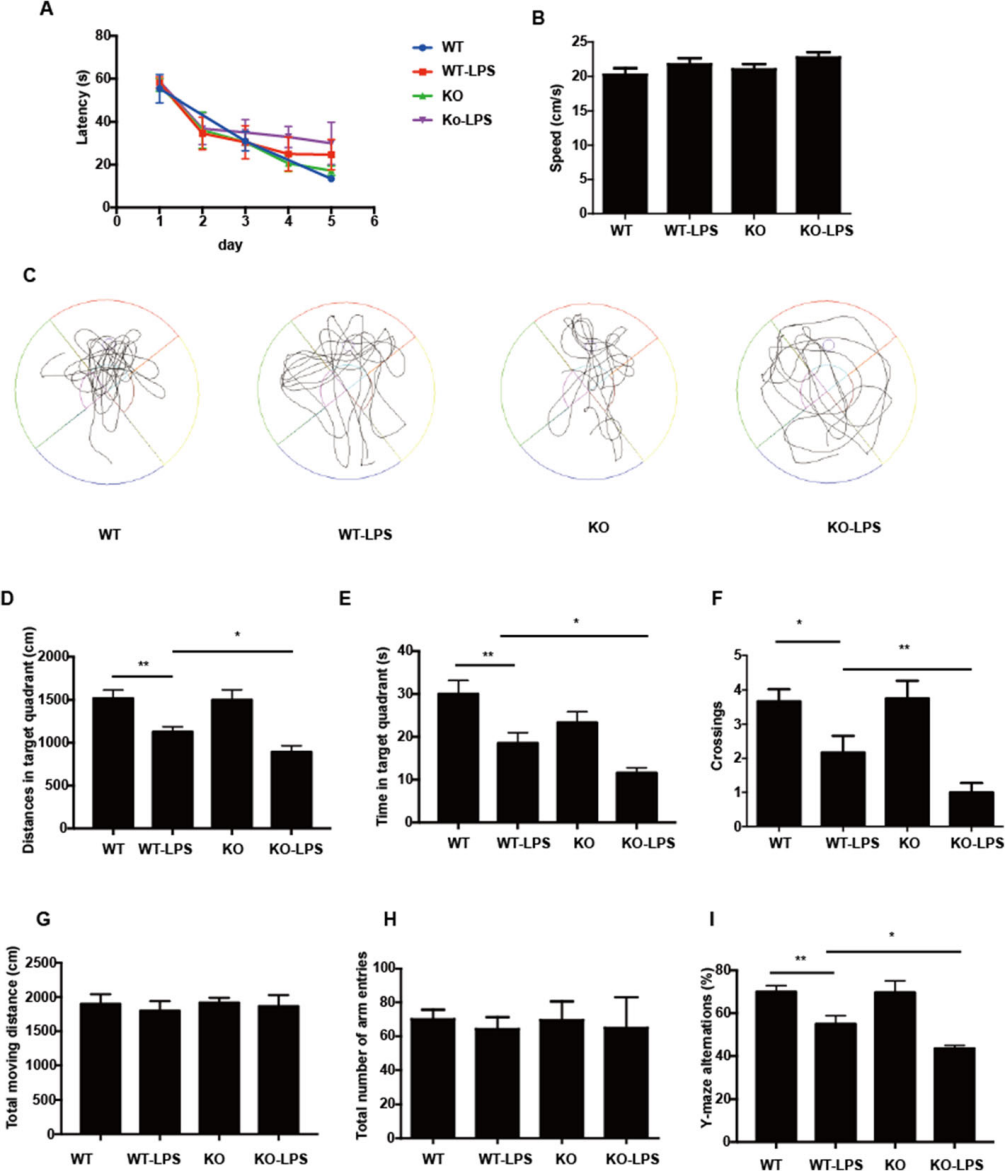
Protective effects of BAP31 in mice with memory deficits caused by cerebral inflammation. The Morris water maze test was performed as described in the “Methods” section. **a** Latency to the platform during spatial working memory testing on days 1, 2, 3, 4, and 5. **b** Swimming speed during probe testing. **c** The swim tracks of the mice during Morris water maze testing. **d** Percentage of the distance traveled in the target quadrant during probe testing. **e** Percentage of the time traveled in the target quadrant during probe testing. **f** Platform-site crossings during probe testing. **g** Spontaneous locomotor activity of mice 7 days after LPS administration. **h**, **i** The Y-maze alternations and total number of arm entries in the Y-maze. *n* = 12 per group for each experiment. Data are expressed as mean ± SEM. *P < 0.05; **P < 0.01; ***P < 0.001 versus control group

Anecdotal evidence suggests that memory deficits caused by neuroinflammation are mediated mostly by the production of proinflammatory cytokines such as TNFα, IL-1β, and COX2 in hippocampus, and it was reported that intracerebroventricular administration of LPS leads to learning and memory deficits. Therefore, we assessed spatial memory formation in age-matched 2-month-old Cre ^LysM-/-^ BAP31^fl/fl^, Cre ^LysM-/-^ BAP31^fl/fl^ + LPS, Cre ^LysM+/-^ BAP31^fl/fl^, and Cre ^LysM+/-^ BAP31^fl/fl^ +LPS mice using the Morris water maze test, including hidden platform training and probe trials. To confirm whether LPS administration resulted in the changes in the behaviors of mice, we performed the open field test. As shown in Fig. 10g, no significant difference was observed in locomotor performance (total moving distance) 7 days after LPS treatment between the four groups of animals, suggesting that the memory impairment in the LPS group was not a result of reduced activity of the mice.

As expected, the escape latency decreased from the first day to the last day of training in all groups, as illustrated in Fig. 10a, but in the Cre ^LysM+/-^ BAP31^fl/fl^ + LPS group, the escape latency was evidently longer than that in the Cre ^LysM-/-^ BAP31^fl/fl^ + LPS groups at day 3 (35.04 ± 4.27 s versus 30.40 ± 5.47 s), day 4 (32.91 ± 3.49 s versus 24.99 ± 5.62 s), and day 5 (29.98 ± 6.97 s versus 24.61 ± 5.01 s). In the probe test, the average speed was not significantly different between the four groups (Fig. 10b); however, the Cre ^LysM+/-^ BAP31^fl/fl^ +LPS groups had significantly decreased the numbers of plate crossings (1.00 ± 0.28 versus 2.16 ± 0.49) in the target quadrant compared with that of the Cre ^LysM-/-^ BAP31^fl/fl^ + LPS group (Fig. 10f). Furthermore, the Cre ^LysM+/-^ BAP31^fl/fl^ + LPS group traveled a shorter distance (892.80 ± 73.66 cm versus 1128.27 ± 59.87 cm) and spent less time (11.60 ± 1.22 s versus 18.59 ± 2.44 s) in the target quadrant (Fig. 10d, e). The swim tracks of the mice during Morris water maze testing are shown in Fig. 10c. These results were supported by Y-maze testing (Fig. 10h, i). The Y-maze alternations were significantly reduced (55.49 ± 3.82 versus 43.56 ± 1.40) in Cre ^LysM+/-^ BAP31^fl/fl^ mice treated with LPS compared with those of the Cre ^LysM-/-^ BAP31^fl/fl^ mice treated with LPS. The total number of arm entries in the Y-maze was also measured between groups, and no significant differences were detected. The above results indicated that upon LPS administration, Cre^LysM+/-^ BAP31^fl/fl^ mice showed severe deficits in spatial memory formation. Therefore, BAP31 may have a crucial role in microglial protection of spatial memory against neuroinflammation.

## Discussion

There is an emerging emphasis on the role of neuroinflammation in driving Alzheimer’s disease progression [33], with mounting evidence implicating a central role of microglia in Alzheimer’s disease, which is characterized by the production of inflammatory mediators that contribute to disease progression and severity. In a clinical study, AD patients had a higher number of activated microglia with an altered phenotype [32]. In AD patients, protein-coding changes are in genes that are highly expressed in microglia and are associated with the innate immune response [34], which provides additional evidence that the microglia-mediated innate immune response contributes directly to AD development. In this study, we first demonstrate that BAP31 deficiency exacerbated cognitive dysfunction induced by cerebroventricular administration of LPS. The protective effect of BAP31 in mice may be relevant to its anti-inflammatory activity by modulating the protein level of IRAK1. High-levels of IRAK1 protein influences the activation of TAK1 upstream of IKK and significantly enhance the NF-κB and MAPK proinflammatory microglial response. Previous studies have identified NF-κB as a major regulator of neuroinflammation [35, 36], which is an increasingly critical participant in the pathology of neurodegenerative diseases, highlighting a critical role of BAP31 in regulating immune responses in the CNS.

Interleukin-1 receptor-associated kinase family members are key mediators in toll-like receptor and interleukin-1 signaling pathways, through which they regulate innate immunity and inflammation [37, 38]. Evidence suggests that IRAKs play essential roles in the pathophysiology of cancer and metabolic and inflammatory diseases [39–41]; IRAK1-deficient mice have reduced liver and kidney damage when treated with LPS compared with that of WT mice [42]; IRAK1 deficiency impacts multiple TLR-dependent pathways and decreases early cytokine responses following polymicrobial sepsis [43], highlighting that IRAK1 inhibition has potential therapeutic benefits. IRAK1 functions as a critical link in the signaling cascade initiated by the binding of ligands to IL-1R and TLRs [44, 45]. The expression of IRAK1 is regulated by translation and posttranscriptional mechanisms [46, 47]. Posttranslational proteolytic degradation via the ubiquitin-proteasome pathway is thought to be one of the major mechanisms of IRAK1 regulation. BAP31 can regulate the degradation of some fast-degrading proteins through the ubiquitin-proteasome pathway. Our studies demonstrated that BAP31 deficiency upregulates the protein level of IRAK1, which has been shown to increase NF-κB activity, and the expression of its target genes, including IL-1β, TNFα, and COX2 [48, 49].

Our previous research has reported that BAP31 promotes the degradation of CFTRDF508 via the Derlin-1 complex [36], and BAP31 regulates p27^kip1^ proteasome degradation [50], which serves as a sorting factor that controls the fate of its client proteins, mediating the subsequent export, retention, degradation or survival. In this study, our data suggest that BAP31 deficiency upregulates the protein level of IRAK1, and therefore enhances microglial responsiveness, whereas the mechanism by which BAP31 regulates IRAK1 still needs further study.

We first showed that BAP31 deficiency improved the LPS-induced pro-inflammatory cytokine production in the microglial BV2 cell line. The expression levels of IL-1β, TNFα, COX2, and iNOS are detrimentally related to the progression of neurodegenerative diseases [51–54]. IL-1β increases inflammatory gene expression and NF-κB activation in primary murine-mixed glia, enriched astrocytes, and BMEC cultures, which is harmful to neurons [55]. Previous studies suggest that modulation of TNFα with small molecule inhibitors is safe and effective with the potential for long-term prevention and treatment of Alzheimer’s disease [56], nitric oxide produced via iNOS upregulation in activated microglia can promote neurodegeneration [57], and some molecules and compounds can play a protective role by inhibiting iNOS expression in the brain of AD [57, 58]. These results thus provide implications that BAP31 might exhibit modulatory effects on neuroinflammation in neurodegenerative diseases such as AD.

BAP31 is a resident, and ubiquitously expressed ER protein [59]. In the present study, the specific knockdown of BAP31 was observed in the microglia of mice using the Cre/loxP system [60–64]. The Morris water maze (MWM) test and Y-maze were chosen as robust and reliable tests that are critically related to hippocampal-dependent memory [65–67]. Consistent with the previously reported results, intracerebroventricular administration of LPS causes cognitive impairment, especially a deficit in short-term memory retention [68, 69]. Then, we examined whether BAP31 deficiency modifies cognitive performance, LPS-induced cognitive deficits, shown a decrease in the rate of spontaneous alternation during the Y-maze and a decrease in platform crossing and target quadrant occupancy during the MWM, suggesting that neuroinflammation contributes to the memory deficits. All these behavioral changes deteriorated with BAP31 deficiency. Our present results are consistent with our previous reports that BAP31 plays an essential role in Alzheimer’s disease by inhibiting the formation of amyloid-β [17]. As expected, Iba1-positive cells significantly increased in the CA1 and DG regions of the hippocampus after challenge with LPS [70, 71], and BAP31 deficiency significantly increased the number of Iba1-positive microglia in mice. Moreover, we demonstrated that intracerebroventricular injection of LPS induced increased IL-1β, TNFα, iNOS, and COX2 levels in the mouse brain 6 h after the injection, consistent with the results from the BV2 cells that BAP31 deficiency exacerbated the expression of these cytokines.

As IL-1β and TNFα are implicated in the survival of neurons, their production may jointly cause neurological abnormalities or neuronal death in the hippocampus, deteriorate spatial memory, and destabilized behavior in BAP31-deficient mice. Therefore, we investigated the effect of BAP31 on the hippocampal neurons following LPS-induced neuroinflammation, and the results showed that the number of NeuN-positive cells were decreased in the LPS groups [72, 73]. As expected, BAP31 deficiency significantly impaired hippocampal neurons in the toxic environment induced by LPS. This result explains why BAP31 deletion could exacerbate learning and memory impairment induced by LPS, and the therapeutic potential of attenuated inflammation signaling in AD is highlighted in studies showing reduced neuron degradation in mice.

In summary, inflammatory mediators that result from NF-κB and AP-1 activation are likely involved in mediating neuronal loss and cognitive impairment. These data are consistent with the hypothesis that BAP31 deficiency exacerbates learning and memory capabilities by mediating a relatively more harmful chronic inflammatory tissue response. This key role of the BAP31 in LPS-mediated inflammatory responses suggests that a therapeutic treatment that blocks the activity of LPS-derived cytokines might effectively interfere with the progression of memory deficits caused by neuroinflammation.

## Conclusions

The findings in the present study suggest the vital role of BAP31 as an immunomodulatory molecule in neuroinflammation. We found that BAP31 deficiency can significantly exacerbate neuroinflammation by enhancing the protein level of IRAK1. In turn, knockdown of IRAK1 by siRNA leads to decreased translocation and transcriptional activity of NF-κB p65 and c-Jun in shBAP31 cells. BAP31 deficiency exacerbates memory deficits caused by experimental cerebral inflammation. Here, we provide the first evidence that BAP31 is involved in regulating inflammatory cytokine production and has a protective effect in learning memory deficits caused by neuroinflammation.

## Supplementary information

**Additional file 1.** (a) The protein level of BAP31 in scramble and shBAP31 BV2 cells by Western bloting for RT-PCR assay. Representative Western blots showing the expression levels of BAP31 and β-actin. The intensity of BAP31 protein was quantified and shown as the ratio to control after normalization by β-actin. (b) The protein level of BAP31 in scramble and shBAP31 BV2 cells by Western bloting for ELISA assay. Representative Western blots showing expression levels of BAP31 and β-actin. The intensity of BAP31 protein was quantified and shown as the ratio to control after normalization by β-actin. (c) The protein level of BAP31 in scramble and shBAP31 Raw264.7 cells. Representative Western blots showing expression levels of BAP31 and β-actin. The intensity of BAP31 protein was quantified and shown as the ratio to control after normalization by β-actin. All the data are indicated as Mean ± SEM of three independent experiments. *P<0.05, **P<0.01, ***P<0.001 versus control group.

**Additional file 2.** (a, b) Scramble and shBAP31 HEK293T cells were cotransfected with NF-κB luciferase reporter plasmid or AP-1 luciferase reporter plasmid and pRL-SV40-C plasmid for 48 h, p65 (a) and c-Jun (b) reporter activity were analyzed following treatment with TNFα for 8 h. All the data are indicated as Mean ± SEM. of three independent experiments. *P<0.05, **P<0.01, ***P<0.001 versus control group.

**Additional file 3.** BAP31 deficiency does not affect the mRNA expression of TLR4 and MyD88. The mRNA levels of TLR4 and MyD88 in scramble and shBAP31 were analyzed by RT-PCR. All the data are indicated as Mean ± SEM of three independent experiments. *P<0.05, **P<0.01, ***P<0.001 versus control group.

**Additional file 4.** Effect of BAP31 deficiency on the protein level of MyD88 in BV2 cells. (a, b) Scramble and shBAP31 BV2 cells were treated with LPS for 0, 5 and 15 min. The intensity of MyD88 protein was quantified and shown as the ratio to control after normalization to β-actin. All the data are indicated as Mean ± SEM of three independent experiments. *P<0.05, **P<0.01, ***P<0.001 versus control group.

**Additional file 5.** The protein level of BAP31 in primary WT and KO microglia cells. Representative Western blots showing expression levels of BAP31 and β-actin. The intensity of BAP31 protein was quantified and shown as the ratio to control after normalization to β-actin. All the data are indicated as Mean ± SEM of three independent experiments. *P<0.05, **P<0.01, ***P<0.001 versus control group.

**Additional file 6.** The protein level of BAP31 in scramble and shBAP31 HEK293T cells. Representative Western blots showing expression levels of BAP31 and β-actin. The intensity of BAP31 protein was quantified and shown as the ratio to control after normalization by β-actin. All the data are indicated as Mean ± SEM of three independent experiments. *P<0.05, **P<0.01, ***P<0.001 versus control group.

**Additional file 7.** Inhibiting IRAK1 protein alleviates the LPS-induced translocation of c-Jun and p65 in shBAP31 cells. (a) Scramble and shBAP31 BV2 cells were treated with IRAK1 inhibitor for 48 h, followed by stimulation with LPS for 30 min. The cytosolic and nuclear fractions were analyzed by Western blotting with antibodies to c-Jun, p65, histone and β-actin. (b) Immunoblots for c-Jun and p65 in cytosolic fractions were quantified and normalized to β-actin protein; immunoblots for c-Jun and p65 in nuclear fractions were quantified and normalized to histone protein. All the data are indicated as Mean ± SEM of three independent experiments. *P<0.05, **P<0.01, ***P<0.001 versus control group.

**Additional file 8.** Schematic illustration of the mouse genomic locus showing the insertion of the targeted allele flanked with LoxP sites (upper) and the deleted allele after Cre recombination (lower).

**Additional file 9.** Microglial BAP31 protein was knocked down in KO mice. Immunofluorescence staining of BAP31 (green) and Iba1 (red) was performed in the hippocampus of the WT and KO groups. Scale bar = 50 μm. n = 12 per group for each experiment.

**Additional file 10: **Effect of BAP31 deficiency on the protein level of IRAK1 in BV2 cells. Scramble and shBAP31 BV2 cells were treated with LPS for 0 min, 5 min, 15 min, 30 min, 1 h, and 2 h. (a) Representative Western blots showing the expression levels of IRAK1. The intensity of protein bands of IRAK1(b) was quantified and shown as the ratio to control after normalization to β-actin. All the data are indicated as Mean ± SEM of three independent experiments. *P<0.05, **P<0.01, ***P<0.001 versus control group.

## Data Availability

All data generated or analyzed during this study are included in this published article and its supplementary information files.

## Abbreviations

BAP31: B cell receptor-associated protein 31
CNS: Central nervous system
COX2: Cyclooxygenase-2
DMSO: Dimethyl sulfoxide
ELISA: Enzyme-linked immune sorbent assay
ER: Endoplasmic reticulum
Iba1: Ionized calcium binding adapter molecule 1
IL-1β: Interleukin-1β
iNOS: Inducible nitric oxide synthase
IRAK1: IL-1 receptor associated kinase
LPS: Lipopolysaccharide
MAPK: Mitogen-activated protein kinase
MTT: 3-(4,5-dimethylthiazol-2-yl)-2,5-diphenyltetrazolium bromide
MWM: Morris water maze
MyD88: Myeloid differentiation factor 88
NF-κB: Nuclear factor kappa B
OFT: Open field test
RT-PCR: Reverse transcription quantitative real-time polymerase chain reaction
TLR4: Toll-like receptor 4
TNF-α: Tumor necrosis factor α
