# Supplementary figures and images for: BAP31 regulates IRAK1-dependent neuroinflammation in microglia
Source: J Neuroinflammation. 2019 Dec 28;16:281. doi: 10.1186/s12974-019-1661-7 (PMC6935200; doi:10.1186/s12974-019-1661-7)

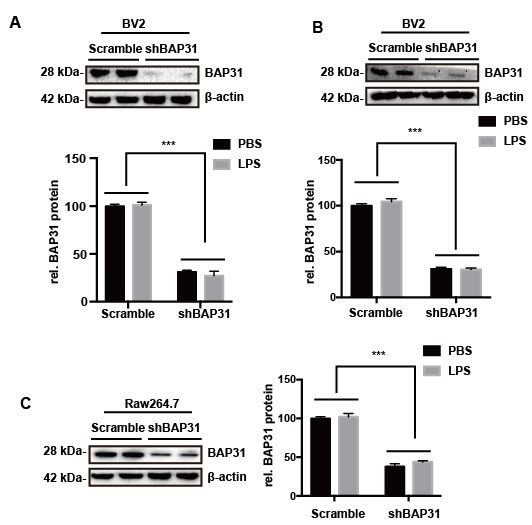

Supplement: Supplementary file 1 — Additional file 1. (a) The protein level of BAP31 in scramble and shBAP31 BV2 cells by Western bloting for RT-PCR assay. Representative Western blots showing the expression levels of BAP31 and β-actin. The intensity of BAP31 protein was quantified and shown as the ratio to control after normalization by β-actin. (b) The protein level of BAP31 in scramble and shBAP31 BV2 cells by Western bloting for ELISA assay. Representative Western blots showing expression levels of BAP31 and β-actin. The intensity of BAP31 protein was quantified and shown as the ratio to control after normalization by β-actin. (c) The protein level of BAP31 in scramble and shBAP31 Raw264.7 cells. Representative Western blots showing expression levels of BAP31 and β-actin. The intensity of BAP31 protein was quantified and shown as the ratio to control after normalization by β-actin. All the data are indicated as Mean ± SEM of three independent experiments. *P<0.05, **P<0.01, ***P<0.001 versus control group. [file 12974_2019_1661_MOESM1_ESM.png]

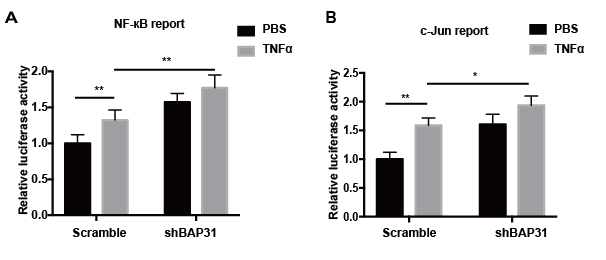

Supplement: Supplementary file 2 — Additional file 2. (a, b) Scramble and shBAP31 HEK293T cells were cotransfected with NF-κB luciferase reporter plasmid or AP-1 luciferase reporter plasmid and pRL-SV40-C plasmid for 48 h, p65 (a) and c-Jun (b) reporter activity were analyzed following treatment with TNFα for 8 h. All the data are indicated as Mean ± SEM. of three independent experiments. *P<0.05, **P<0.01, ***P<0.001 versus control group. [file 12974_2019_1661_MOESM2_ESM.png]

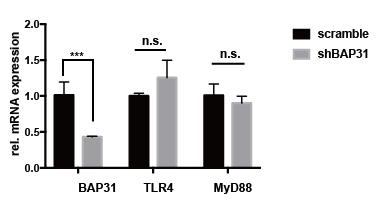

Supplement: Supplementary file 3 — Additional file 3. BAP31 deficiency does not affect the mRNA expression of TLR4 and MyD88. The mRNA levels of TLR4 and MyD88 in scramble and shBAP31 were analyzed by RT-PCR. All the data are indicated as Mean ± SEM of three independent experiments. *P<0.05, **P<0.01, ***P<0.001 versus control group. [file 12974_2019_1661_MOESM3_ESM.png]

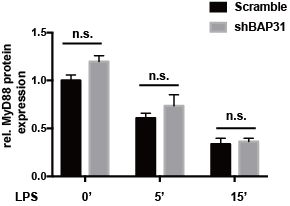

Supplement: Supplementary file 4 — Additional file 4. Effect of BAP31 deficiency on the protein level of MyD88 in BV2 cells. (a, b) Scramble and shBAP31 BV2 cells were treated with LPS for 0, 5 and 15 min. The intensity of MyD88 protein was quantified and shown as the ratio to control after normalization to β-actin. All the data are indicated as Mean ± SEM of three independent experiments. *P<0.05, **P<0.01, ***P<0.001 versus control group. [file 12974_2019_1661_MOESM4_ESM.png]

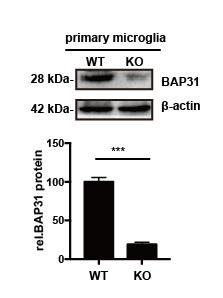

Supplement: Supplementary file 5 — Additional file 5. The protein level of BAP31 in primary WT and KO microglia cells. Representative Western blots showing expression levels of BAP31 and β-actin. The intensity of BAP31 protein was quantified and shown as the ratio to control after normalization to β-actin. All the data are indicated as Mean ± SEM of three independent experiments. *P<0.05, **P<0.01, ***P<0.001 versus control group. [file 12974_2019_1661_MOESM5_ESM.png]

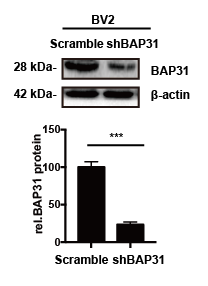

Supplement: Supplementary file 6 — Additional file 6. The protein level of BAP31 in scramble and shBAP31 HEK293T cells. Representative Western blots showing expression levels of BAP31 and β-actin. The intensity of BAP31 protein was quantified and shown as the ratio to control after normalization by β-actin. All the data are indicated as Mean ± SEM of three independent experiments. *P<0.05, **P<0.01, ***P<0.001 versus control group. [file 12974_2019_1661_MOESM6_ESM.png]

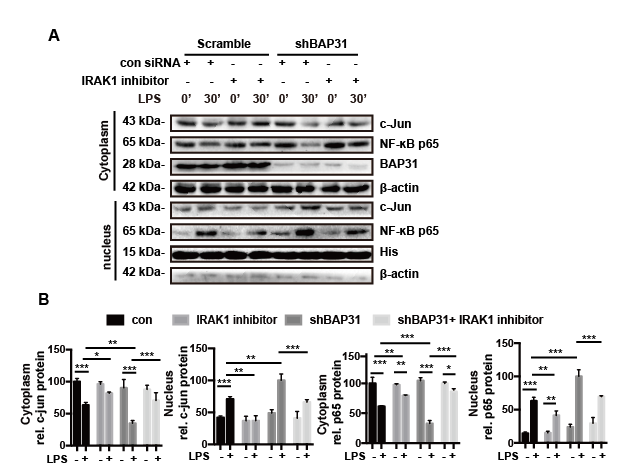

Supplement: Supplementary file 7 — Additional file 7. Inhibiting IRAK1 protein alleviates the LPS-induced translocation of c-Jun and p65 in shBAP31 cells. (a) Scramble and shBAP31 BV2 cells were treated with IRAK1 inhibitor for 48 h, followed by stimulation with LPS for 30 min. The cytosolic and nuclear fractions were analyzed by Western blotting with antibodies to c-Jun, p65, histone and β-actin. (b) Immunoblots for c-Jun and p65 in cytosolic fractions were quantified and normalized to β-actin protein; immunoblots for c-Jun and p65 in nuclear fractions were quantified and normalized to histone protein. All the data are indicated as Mean ± SEM of three independent experiments. *P<0.05, **P<0.01, ***P<0.001 versus control group. [file 12974_2019_1661_MOESM7_ESM.png]

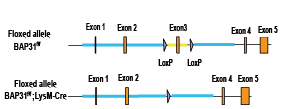

Supplement: Supplementary file 8 — Additional file 8. Schematic illustration of the mouse genomic locus showing the insertion of the targeted allele flanked with LoxP sites (upper) and the deleted allele after Cre recombination (lower). [file 12974_2019_1661_MOESM8_ESM.png]

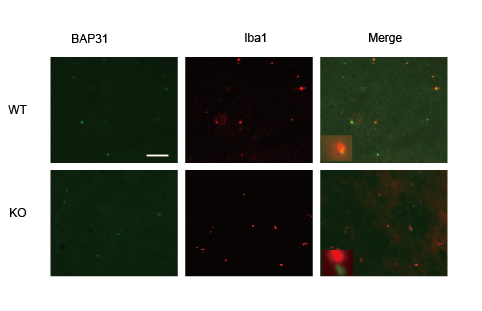

Supplement: Supplementary file 9 — Additional file 9. Microglial BAP31 protein was knocked down in KO mice. Immunofluorescence staining of BAP31 (green) and Iba1 (red) was performed in the hippocampus of the WT and KO groups. Scale bar = 50 μm. n = 12 per group for each experiment. [file 12974_2019_1661_MOESM9_ESM.png]

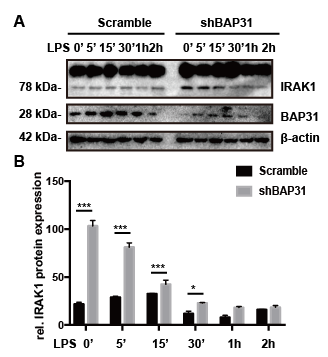

Supplement: Supplementary file 10 — Additional file 10: Effect of BAP31 deficiency on the protein level of IRAK1 in BV2 cells. Scramble and shBAP31 BV2 cells were treated with LPS for 0 min, 5 min, 15 min, 30 min, 1 h, and 2 h. (a) Representative Western blots showing the expression levels of IRAK1. The intensity of protein bands of IRAK1(b) was quantified and shown as the ratio to control after normalization to β-actin. All the data are indicated as Mean ± SEM of three independent experiments. *P<0.05, **P<0.01, ***P<0.001 versus control group. [file 12974_2019_1661_MOESM10_ESM.png]
